# Two cases of microscopic replantation of auricular dissection in different planes: Case report

**DOI:** 10.1097/MD.0000000000044826

**Published:** 2025-10-17

**Authors:** Sen Zhao, Fengrui Liu

**Affiliations:** a Taiyuan Micro Hand Surgery Hospital, Taiyuan, China; b The First Hospital of Shanxi Medical University, Taiyuan, China.

**Keywords:** auricular avulsion, case report, microsurgical replantation, vascular reconstruction

## Abstract

**Rationale:**

Auricular lacerations are commonly encountered in clinical practice, and such injuries can cause significant damage to both the appearance of the face and the emotional well-being of the individual. This case report presents the clinical experience and key considerations of microreplantation of auricular lacerations in different planes, aiming to provide a reference for clinical treatment.

**Patient concerns:**

This study analyzed 6 cases of auricular avulsion microsurgical replantation, all achieving excellent clinical outcomes. After systematically evaluating various anatomical planes, long-term follow-up (up to 6 years), and different surgical techniques (anterograde vs retrograde replantation), we present 2 representative cases. All patients underwent meticulous preoperative assessment of the avulsed auricles followed by successful microsurgical replantation. Postoperative results demonstrated well-survived replanted auricles without significant pigmentation changes, near-complete sensory recovery, and fully preserved auditory function.

**Diagnoses:**

Due to various unpredictable causes such as trauma, the auricle can undergo avulsion at different planes, which is diagnosed through physical examination. The examination revealed exposed and fractured auricular cartilage at both the proximal and distal ends, with active bleeding points at the proximal ear and partial contusions at the distal auricle.

**Interventions:**

According to the avulsion plane of the auricle, followed by meticulous debridement of both the proximal and distal segments under microscopy, employing a carpet-like excisional technique. Depending on the vascular anatomy and extent of tissue loss, either anterograde or retrograde microvascular replantation was performed. Long-term follow-up was conducted to evaluate outcomes.

**Outcomes:**

Both replanted auricles survived completely. Postoperative follow-up ranged from 6 months to 7 years. One patient developed a venous crisis leading to scab detachment and mild auricular collapse. Another case exhibited partial skin loss at the junction of the auricle and earlobe. At 10 weeks postoperatively, the second patient underwent a Z-plasty revision under local infiltration anesthesia to address the defect. At final follow-up, all replanted auricles showed no significant pigmentation, near-normal sensory recovery, and preserved auditory function.

**Lessons:**

Intraoperative exploration of vascular integrity based on the level of injury is essential, allowing for precise microvascular anastomosis under microscopy. Adequate vascular anastomosis significantly promotes the survival of the replanted auricle.

## 1. Introduction

Auricular avulsion injuries are not uncommon in clinical practice and can result in significant aesthetic disfigurement and psychological trauma. Therefore, timely and effective intervention is essential to maximize restoration of facial appearance and minimize the physical and emotional impact on patients.^[1]^ Microsurgical replantation with vascular anastomosis remains the optimal approach for repairing complete auricular amputations.^[2]^ Various surgical techniques have been reported in the literature, including precise in situ reattachment, auricular cartilage replantation covered with local fascial flaps, cartilage burial in the abdominal wall or postauricular subcutaneous space followed by staged reconstruction, and auricular reconstruction using costal cartilage grafts. However, these alternative methods often involve extensive trauma and require multiple surgical procedures.^[3]^ In 2010, Koshima et al introduced the concept of supermicrosurgery, characterized by reduced invasiveness and faster postoperative recovery. The present study applies and embodies the principles of microsurgery in clinical practice.^[4]^ From March 2014 to March 2024, Taiyuan Microsurgery Hospital treated multiple patients with auricular avulsion injuries involving various planes of detachment, ranging in age from 26 to 58 years. The majority of these cases underwent microsurgical replantation with vascular anastomosis, all of which yielded satisfactory outcomes.

In this report, we present 2 representative cases of total auricular amputation. These cases highlight different levels of avulsion and demonstrate the advantages of varying surgical techniques. Long-term follow-up, up to 6 years in some instances, revealed favorable outcomes in terms of both sensory recovery and facial aesthetics. These cases underscore the clinical value of microsurgical auricular replantation and may serve as a useful reference for clinicians managing similar injuries.

## 2. Typical case report

### 2.1. Case 1

#### 2.1.1. Basic situation

A 33-year-old male patient was admitted to the emergency department following a traffic accident that occurred 4 hours prior, resulting in a traumatic avulsion and complete detachment of the right auricle. The patient complained of pain and bleeding at the injury site but denied dizziness, nausea, vomiting, or other systemic symptoms.

Initial management at a local hospital included simple wound dressing for hemostasis, intramuscular injection of tetanus antitoxin, and cranial computed tomography, which revealed no significant abnormalities.

Upon admission, the patient was alert and oriented, with stable appetite, sleep, and normal bowel and bladder function. He had no history of loss of consciousness. Vital signs were as follows: body temperature 37.8°C, pulse 98 bpm, respiratory rate 20 breaths/min, and blood pressure 133/81 mm Hg.

**Figure 1.**
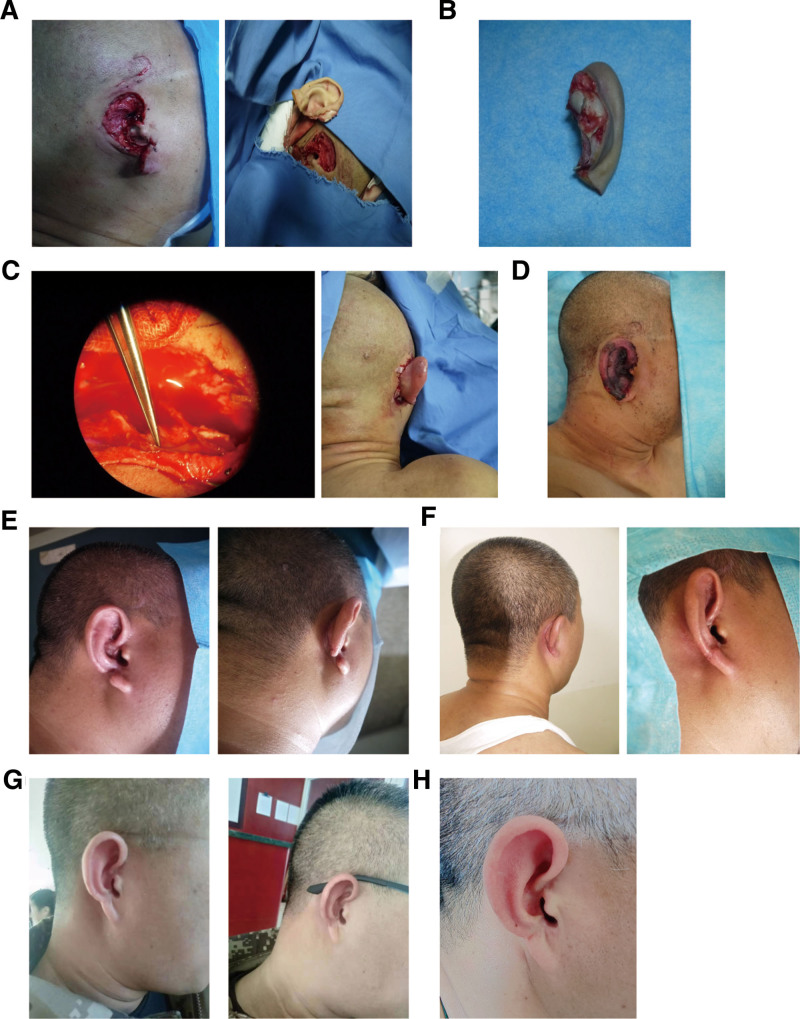
Case 1, 33-year-old male. (A) Preoperative injury; (B) preoperative microscopic exploration of the ear vessels; (C) intraoperative anastomosis of the vessels; (D) postoperative condition; (E) 6 weeks postoperatively; (F) 12 weeks postoperatively; (G) 2 years postoperatively; (H) 6 years postoperatively.

Physical examination revealed a complete avulsion of the right auricle from its root, with only a small remnant of earlobe tissue proximally attached. Both proximal and distal wound edges were irregular, and shattered cartilage was exposed on both sides of the injury. Active bleeding was observed at the proximal stump. The distal auricular segment showed contusions and partial skin loss. Additionally, a laceration of the scalp was noted superior to the auricle, with partial degloving and soft tissue defect of the auricular skin (Fig. 1A, B).

#### 2.1.2. Treatment

Following admission, comprehensive preoperative preparation was initiated. After multidisciplinary consultation, the patient underwent surgery under general anesthesia. Preoperative hair removal was performed around the injured ear, and the external auditory canal was gently packed with sterile cotton to prevent the ingress of blood, disinfectants, and irrigation fluid during the lateral decubitus positioning. The patient was placed in a lateral decubitus position with careful adjustment of the surgical bed to ensure a comfortable and stable posture for the prolonged and meticulous microsurgical procedure. The surgical area, particularly the head and face, was optimized for access. The wound was repeatedly irrigated with hydrogen peroxide, normal saline, and diluted povidone-iodine solution. Standard surgical disinfection and draping were performed.

Intraoperatively, thorough debridement was carried out under microscopy. Irregular wound edges were trimmed, and fragmented auricular cartilage was excised. Limited sharp dissection was performed between the skin and cartilage based on the level of avulsion to facilitate precise cartilage repair. Carpet-like meticulous debridement of both the proximal and distal wound surfaces was completed under the microscope. When necessary, skin incisions were extended to expose vessels and nerves. Microvascular and neural structures were marked for repair.

**Figure 2.**
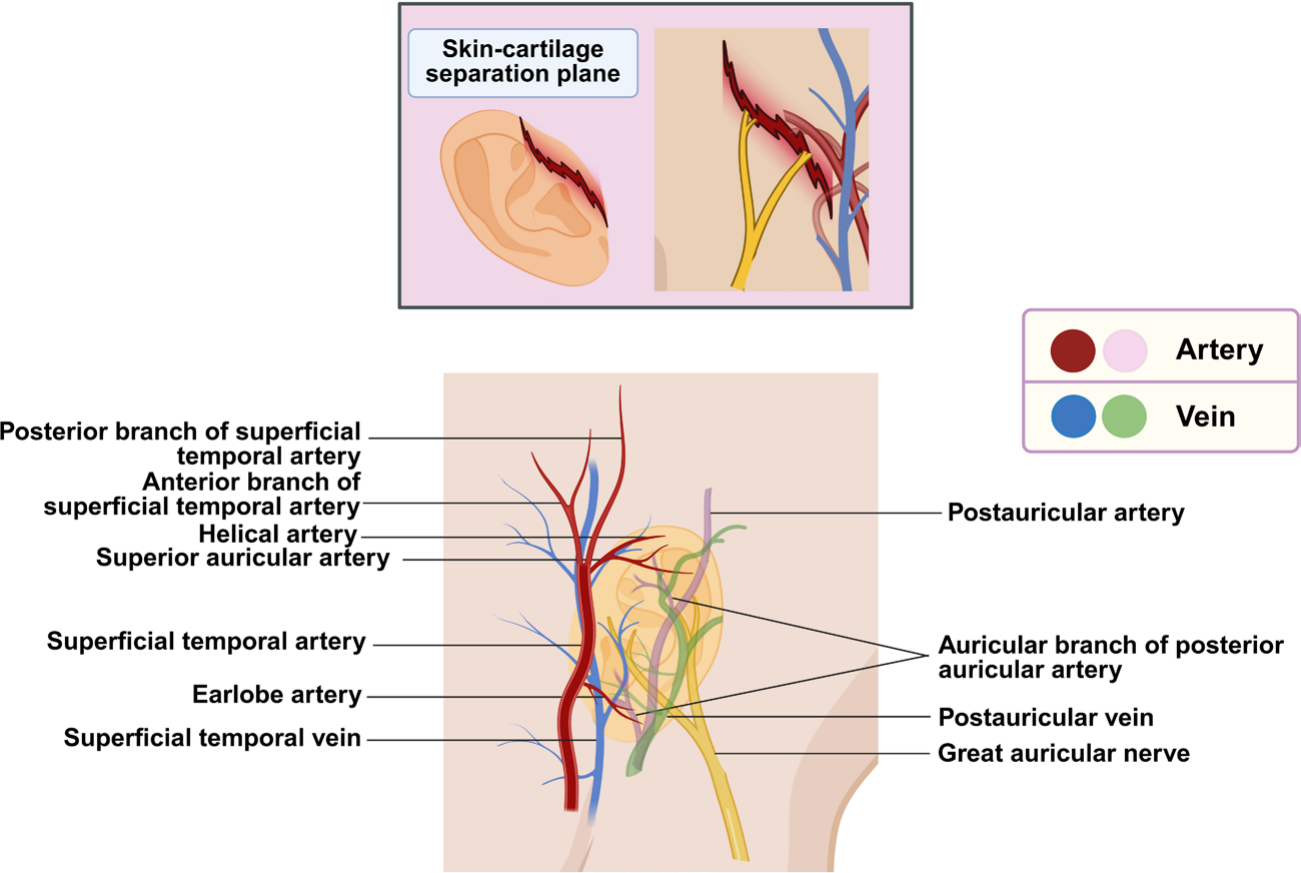
Schematic representation of arterial and venous networks supplying the auricle.

Auricular cartilage was precisely reconstructed using 6-0 absorbable sutures. The auricle was completely avulsed at the level of the auricular root and external auditory canal. An antegrade replantation approach was adopted. Under microscopic visualization, the posterior auricular artery’s middle and inferior branches, 1 posterior auricular vein, 1 accompanying vein of the inferior auricular branch, and a branch of the great auricular nerve were successfully anastomosed. Skin closure was performed with interrupted sutures. Several small rubber drains were placed. Postoperatively, blood crusts were gently wiped with warm saline swabs. Multiple small incisions were made to allow for bloodletting, and subcutaneous injections of heparin sodium solution were administered (Fig. 2). Wound care included povidone-iodine swabbing and loose sterile dressing to avoid circumferential compression. A fenestrated gauze pad was placed over the auricle and fixed with oil gauze to facilitate observation.

Postoperative monitoring showed the replanted auricle to be red, warm, with normal tension and capillary refill. The patient received subcutaneous heparin injections at 5000 U daily for 7 days. Activated partial thromboplastin time and other coagulation parameters were closely monitored. On postoperative day 3, localized scattered hemorrhagic blisters developed, which were managed conservatively without rupture. The blisters resolved within 1 week. By postoperative week 2, auricular viability was confirmed. A minor area of skin necrosis was observed at the junction of the auricle and earlobe. Overall survival of the right auricle was excellent. Two months postoperatively, the patient underwent local Z-plasty revision under infiltrative anesthesia. The wound healed primarily. The patient was followed for 6 years, during which the auricular skin remained rosy, with satisfactory contour, normal sensory recovery, and preserved hearing. The patient reported no cold intolerance or frostbite during winter (Fig. 1C–H).

### 2.2. Case 2

#### 2.2.1. Basic situation

A 42-year-old female patient was admitted to the emergency department 6 hours after a partial auricular avulsion injury caused by a machine-related trauma to the right ear. Since admission, the patient exhibited poor appetite, sleep, and general condition, but maintained normal bowel and bladder function. She had a 2-year history of hypertension, for which she had been taking simvastatin 40 mg once daily. Vital signs on admission were as follows: body temperature 36.5°C, pulse 78 bpm, respiratory rate 19 breaths/min, and blood pressure 155/97 mm Hg. The external auditory canal was patent with no abnormal discharge. No tenderness was noted in the mastoid region, and crude hearing assessment was normal.

Physical examination revealed an obliquely oriented, partially complete avulsion of the right auricle at the level of the external ear. The wound edges on both the proximal and distal sides were irregular, with exposed and partially fragmented auricular cartilage. Active bleeding was observed at the proximal stump, while the distal auricular segment showed contusions.

#### 2.2.2. Treatment

Comprehensive preoperative preparation was initiated upon admission. Following multidisciplinary consultation, surgery was performed under general anesthesia. Preoperative hair removal was conducted around the affected ear, and the external auditory canal was gently packed with sterile cotton to prevent the entry of blood, disinfectants, or irrigation fluid during lateral decubitus positioning. The patient was positioned laterally, with careful adjustment of the operating table to ensure a stable and ergonomically favorable posture for the delicate and time-intensive microsurgical procedure. The surgical field was thoroughly irrigated using hydrogen peroxide, normal saline, and diluted povidone-iodine solution. Standard aseptic preparation and draping were performed.

Under microscopic guidance, thorough debridement and wound edge trimming were carried out. Fragmented auricular cartilage was excised. Limited sharp dissection was performed between the skin and cartilage depending on the avulsion plane, which facilitated accurate cartilage alignment and repair. Carpet-like meticulous debridement of the proximal and distal wound surfaces was performed under microscopy. When necessary, the skin incision was extended to expose neurovascular structures, which were identified and marked for later repair.

Auricular cartilage was reconstructed using 6-0 absorbable sutures. Microscopic exploration revealed 1 fine branch of the superior auricular artery from the superficial temporal artery anterior-superiorly, as well as 1 branch of the posterior auricular artery posterior-inferiorly. One posterior auricular vein and 1 accompanying vein of the posterior auricular artery were also identified. A retrograde replantation approach was employed. Anastomoses were performed between branches of the superficial temporal and posterior auricular arteries, as well as the corresponding veins. A branch of the great auricular nerve was also coapted posteriorly (Fig. 2). The skin was loosely approximated with interrupted sutures, and multiple slender rubber drain strips were placed. Postoperatively, dried blood crusts were gently removed using warm saline swabs. Multiple small incisions were made to facilitate controlled bloodletting, and subcutaneous heparin sodium solution was injected. The wound was disinfected with povidone-iodine swabs and loosely dressed to avoid circular compression. A fenestrated gauze pad was tailored to cradle and secure the auricle, which was covered with oil gauze to allow observation. Postoperatively, the replanted auricle appeared purplish-red. Small incisions were again made to relieve congestion, and the patient received 5000 U of subcutaneous heparin daily for 7 days. Activated partial thromboplastin time and other coagulation indices were monitored, and symptomatic treatments were administered. Dressings were changed regularly, with warm saline swabs used to remove scabs.

**Figure 3.**
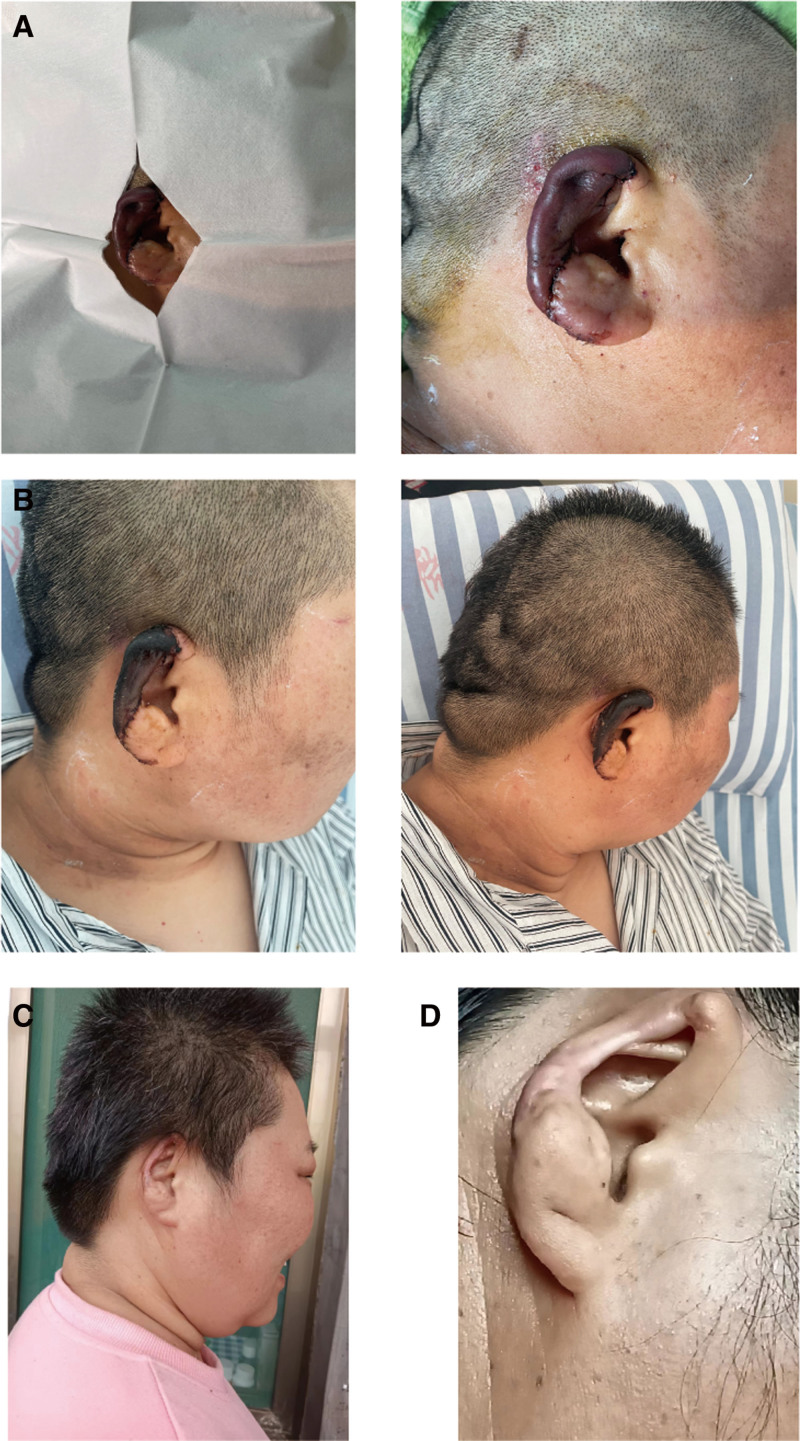
Case 2, female, 42 years old. (A) Postoperative condition; (B) 6 weeks after surgery; (C) 3 months after surgery; (D) 1 year after surgery.

By postoperative week 2, the auricular skin had formed a firm, dry eschar. Following regular wound care and debridement, the eschar detached spontaneously after 6 weeks, and the wound healed. At the 1-year follow-up, mild auricular contraction and collapse were noted, but no significant pigmentation changes were observed. Notably, partial recovery of protective sensation was achieved, hearing function remained preserved, and the patient reported no episodes of frostbite or cold intolerance during the winter season (Fig. 3).

## 3. Discussion

Traumatic auricular avulsion remains a formidable challenge for plastic and reconstructive surgeons. Successful auricular replantation is crucial for restoring both facial sensation and aesthetics.^[5]^ A key determinant of replantation success is the restoration of adequate blood supply, making microvascular anastomosis indispensable in most cases. While several reports have described non-microvascular techniques for auricular reattachment, these methods often carry a high risk of failure.^[6]^ Although successful outcomes have occasionally been observed in non-vascularized auricular replantation, the lack of revascularization significantly compromises tissue survival.^[7,8]^ Therefore, reestablishing vascular perfusion is critical for optimal outcomes.^[9,10]^ This case series demonstrates methods for revascularizing the amputated auricle in different planes of detachment. Intraoperative meticulous debridement under microscopy is essential. Due to the variability in shape and condition of the amputated segments, neurovascular structures must be identified through systematic, carpet-like dissection and appropriately marked. Microsurgical anastomosis of arteries and veins is then performed to ensure tissue viability. The auricular vasculature is complex and highly variable. The extent and pattern of vascular injury depend on the specific trauma involved. This study compares 2 microsurgical approaches – antegrade (orthograde) replantation and retrograde replantation – both of which yielded favorable replantation outcomes. Antegrade replantation involves first anastomosing the veins followed by the arteries, and is generally preferred when proximal arterial supply is intact and vascular anatomy is clearly defined. This technique benefits from physiological blood flow direction, allowing smooth venous return. However, it requires precise vessel matching and a high level of surgical expertise. Retrograde replantation, on the other hand, entails arterial anastomosis before venous repair. This method is especially useful when proximal vessels are disrupted or inaccessible, and when antegrade perfusion is not feasible. Retrograde techniques rely on collateral circulation to reverse the flow and maintain perfusion. While this approach may involve more intraoperative adjustments, it can reduce ischemic risk in anatomically compromised cases. Ultimately, the choice between antegrade and retrograde replantation should be based on the vascular status of the avulsion site and intraoperative findings, emphasizing individualized surgical planning to optimize outcomes.

The auricle’s vascular supply is primarily derived from branches of the superficial temporal artery and the posterior auricular artery.^[11]^ The anterior auricular branch of the superficial temporal artery has a typical diameter of approximately 0.1 mm. The superior and middle branches of the posterior auricular artery measure approximately 0.2 to 0.3 mm in diameter, while the inferior auricular branch is the largest, with a diameter ranging from 0.3 to 0.5 mm. The accompanying veins typically measure an average of 0.65 mm (range: 0.3 mm–2.0 mm). The posterior surface and medial aspect of the auricle are mainly supplied by branches of the posterior auricular artery, which forms multiple interbranch anastomoses. Specifically, the ascending branch of the superior auricular branch anastomoses with the upper auricular branch of the superficial temporal artery. The inferior auricular branch of the posterior auricular artery also forms connections with perforators from the earlobe and the lower auricular branch of the superficial temporal artery. The helical rim receives its blood supply from 2 primary vascular arcades: 1 located on the medial surface and another on the lateral surface of the helix.^[12,13]^ Due to the extremely fine caliber of these vessels – especially the veins, which possess thin and fragile walls – microvascular anastomosis in auricular replantation is technically demanding and constitutes a major challenge.

**Figure 4.**
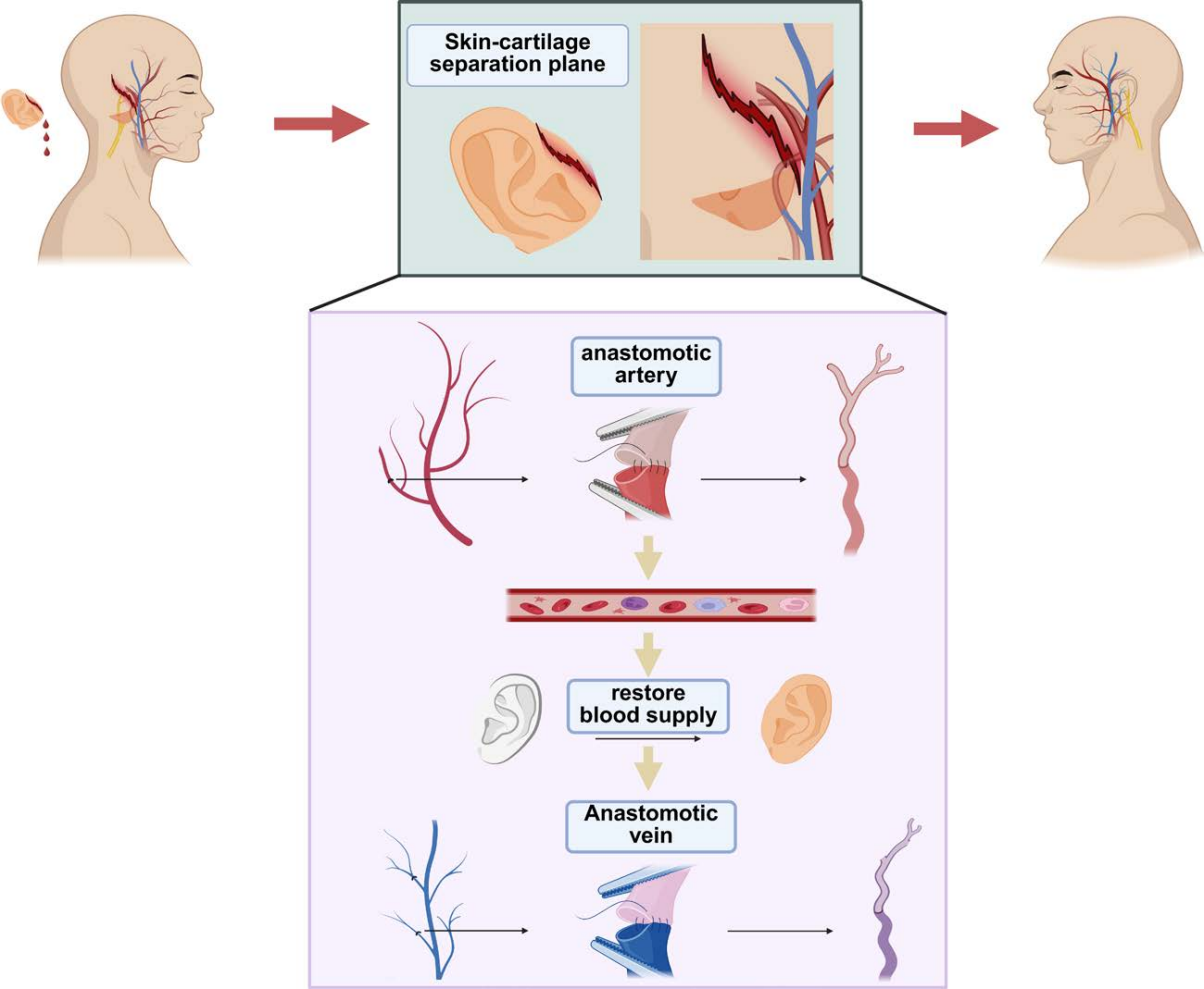
Schematic representation of microsurgical technique for auricular replantation.

In cases involving limited auricular avulsion or where venous identification is difficult, arterial anastomosis may be prioritized.^[14]^ Once arterial flow is reestablished, venous congestion in the distal segment may facilitate the identification and subsequent repair of additional vessels. Based on the anatomical network, successful high-quality anastomosis of even a single branch of the posterior auricular artery can perfuse the entire auricle through interbranch collateral pathways. Intraoperatively, regardless of the vessel caliber, all identifiable arteries and veins should be anastomosed whenever possible to maximize perfusion and enhance auricular viability. Moreover, auricular vessels are extremely delicate and vulnerable to injury. Avulsion often leads to extensive separation between the skin and cartilage, resulting in vessel contusion or transection. Under high-magnification microscopy, painstaking dissection and trimming of vessels to healthy segments is essential to ensure tension-free and patent anastomoses (Fig. 4).

In summary, complete auricular avulsion injuries present with varying severities and morphological patterns depending on the plane and extent of detachment. Tailored surgical strategies should be employed based on the specific characteristics of each injury. Early and meticulous microsurgical debridement is essential, with an emphasis on preserving viable tissue whenever possible. The auricle is composed of thin subcutaneous tissue, with superficial arteries and veins of extremely small caliber, making vessel identification and dissection particularly challenging – especially given the high risk of vascular disruption from avulsion trauma. Nevertheless, microsurgical replantation, guided by detailed knowledge of auricular vascular anatomy, allows for the identification, dissection, and high-quality anastomosis of viable arteries and veins. By selecting the appropriate surgical approach – be it antegrade or retrograde replantation – and executing tension-free, precise vascular anastomoses, surgeons can significantly improve the success rate and overall outcomes of auricular replantation. Ensuring robust revascularization remains the cornerstone of promoting graft survival and achieving both functional and aesthetic restoration.

## 4. Conclusion

The application of microsurgical techniques in auricular replantation has demonstrated favorable therapeutic outcomes, significantly alleviating patients’ psychological distress and aesthetic concerns. Vascular anastomosis remains the cornerstone of successful auricular reattachment. This study presents our clinical experience and surgical strategies for microsurgical replantation across various planes of auricular avulsion, emphasizing key technical considerations. Long-term follow-up further supports the efficacy of these approaches, offering valuable reference data for clinicians managing complex auricular trauma.

## Acknowledgments

The authors would like to thank our department colleagues and these patients for their dedication.

## Author contributions

**Supervision:** Sen Zhao.

**Writing – original draft:** Sen Zhao.

**Writing – review & editing:** Fengrui Liu.
